# Bardet-Biedl Syndrome Presenting With Bifid Epiglottis: A Case Report and Review of Literature

**DOI:** 10.7759/cureus.37849

**Published:** 2023-04-19

**Authors:** Saif A Saif, Suzan S Alzaidi, Abdullah F Alghamdi, Amal A Alharazi, Omar S Almansouri, Rehab Fadag

**Affiliations:** 1 College of Medicine, King Saud Bin Abdulaziz University for Health Sciences, Jeddah, SAU; 2 Otolaryngology, Head and Neck Surgery, King Fahad Armed Forces Hospital, Jeddah, SAU; 3 Medicine and Surgery, King Saud Bin Abdulaziz University for Health Sciences, Jeddah, SAU; 4 Otolaryngology, Head and Neck Surgery, Al-Qunfudah Health Affairs Directorate, Qunfudah, SAU; 5 Histopathology, King Fahad Armed Forces Hospital, Jeddah, SAU

**Keywords:** syndromic bifid epiglottis, review of literature, bardet-beidl syndrome, laurence-moon-bardet-biedl syndrome, bifid epiglottis

## Abstract

Bifid epiglottis is a rare congenital laryngeal anomaly that is most commonly a syndromic rather than an isolated entity. It has been associated with specific syndromes, such as Pallister-Hall syndrome, Bardet-Biedl syndrome, and other related syndromes. Bardet-Biedl syndrome is a rare autosomal-recessive disorder characterized by hand and/or feet polydactyly, obesity, short stature, mental retardation, renal anomalies, and genital anomaly. Here we report a case involving a 25-year-old Saudi male patient who presented with hoarseness of voice since birth with no diurnal or diet association or other associated symptoms. On examination, he was noted to have craniofacial dysmorphism and polydactyly of the right hand and left foot. Fiberoptic nasopharyngolaryngoscopy (NPLS) revealed a laryngeal pedunculated rounded glottic mass and subglottic bulging with expiration and involuting with inspiration along with an abnormal-looking epiglottis having a separate cartilaginous framework with space in-between and bilateral mobile vocal cords. Computed tomography (CT) showed the vocal cord mass and a bifid epiglottis. Other investigations and labs were within normal range. The patient underwent vocal cord mass excision and soft tissue histopathology revealed a benign growth. On follow-up, the patient showed clinical improvement. In conclusion, this is a rare case of bifid epiglottis associated with Bardet-Biedl syndrome, which serves to highlight the significance of such anomalies in any syndromic patient presenting with airway symptoms. Our aim is to add more cases to the literature and to consider it as a differential diagnosis.

## Introduction

Bardet-Biedl syndrome (BBS), a form of Laurence-Moon-Bardet-Biedl syndrome (LMBBS) is an autosomal recessive disorder caused by mutations in BBS genes whose function is related to the primary cilium. To date, mutations in 24 BBS genes have been reported; however, mutations in BBS1, BBS2, and BBS10 account for 50% of BBS cases. The disease-causing gene is identified in 80% of cases. The prevalence of upper airway abnormalities in BBS remains unknown, but laryngeal webs and bifid epiglottis (BE) have been reported. A congenital laryngeal web is an upper airway malformation resulting from incomplete recanalization of the primitive larynx, representing less than 5% of larynx congenital anomalies [1,2]. Moreover, this syndrome is characterized by retinal degeneration, polydactyly, obesity, mental retardation, hypogenitalism, renal dysplasia, and short stature [1]. BBS has an estimated incidence of 1:13,500 in the Middle East, and an incidence of 1:160,000 in the rest of the world, with a male-to-female ratio of approximately 1.3:1 [2]. Although a few reports focused on the presence of otolaryngologic abnormalities associated with LMBBS, among the laryngeal anomalies, acquired lesions secondary to infections are more commonly reported than congenital laryngeal anomalies; laryngomalacia has been reported as the most common congenital lesion, occurring in 60-70% of cases. On the other hand, laryngeal webs (5% of cases), and specifically BE, have been found infrequently. Other otolaryngologic features include language and speech disorders, oral and dental abnormalities, and sensorineural hearing loss [3]. Out of the 24 cases of BE reported in the literature, only nine of them were associated with BBS.

BE has also been rarely reported to be associated with BSS [3]. BE is a rare congenital laryngeal anomaly that has been increasingly associated with genetic syndromes, such as Pallister-Hall, and LMBBS [4-8]. BE has been reported in 40% of patients with Pallister-Hall syndrome and rarely along with other syndromes [5].

We report a case of a 25-year-old male genetically confirmed to have BBS who presented with hoarseness of voice. During the examination, a left vocal cord mass and bifid epiglottis were found. The mass was completely excised and sent to pathology. At the two-week follow-up, the patient showed improvement.

Moreover, we searched English-language literature from 1990 through 2022 via PubMed, Google, and Google Scholar for similar studies using the following search keywords: "Bardet-Biedl syndrome", "bifid epiglottis", and "syndromic bifid epiglottis". We aimed to collect all reported cases of BE associated with BBS in order to emphasize important points of this condition, including demographics, presentation, examination findings, radiological findings, histopathology results, and treatment.

## Case presentation

**Figure 1:**
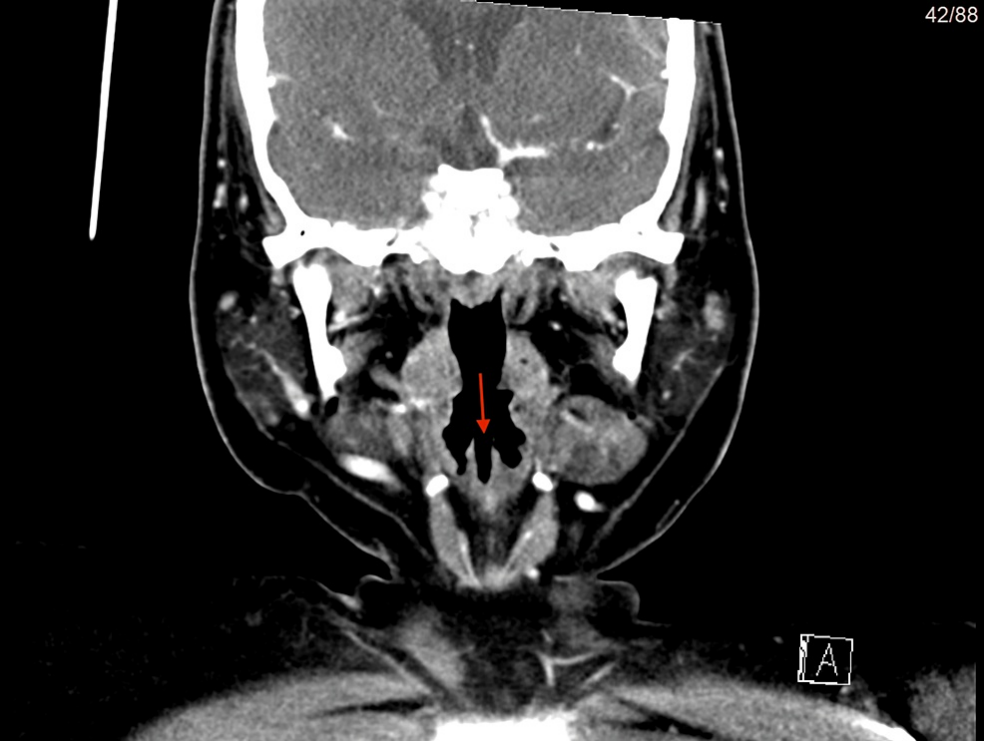
(CT scan) in coronal view The red arrow shows the bifid epiglottis with a cleft confined only to the upper one-third of the epiglottis, not reaching the base.

**Figure 2:**
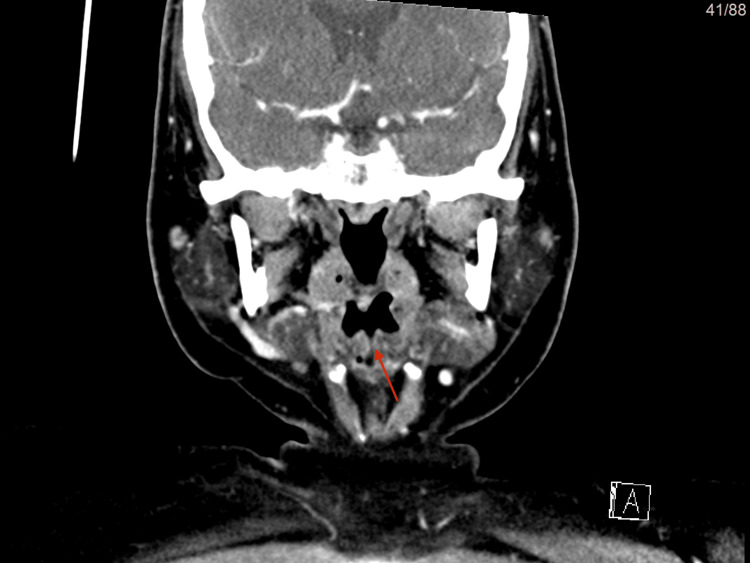
(CT scan) in this coronal view The red arrow shows the bifid epiglottis in a more posterior view.

**Figure 3:**
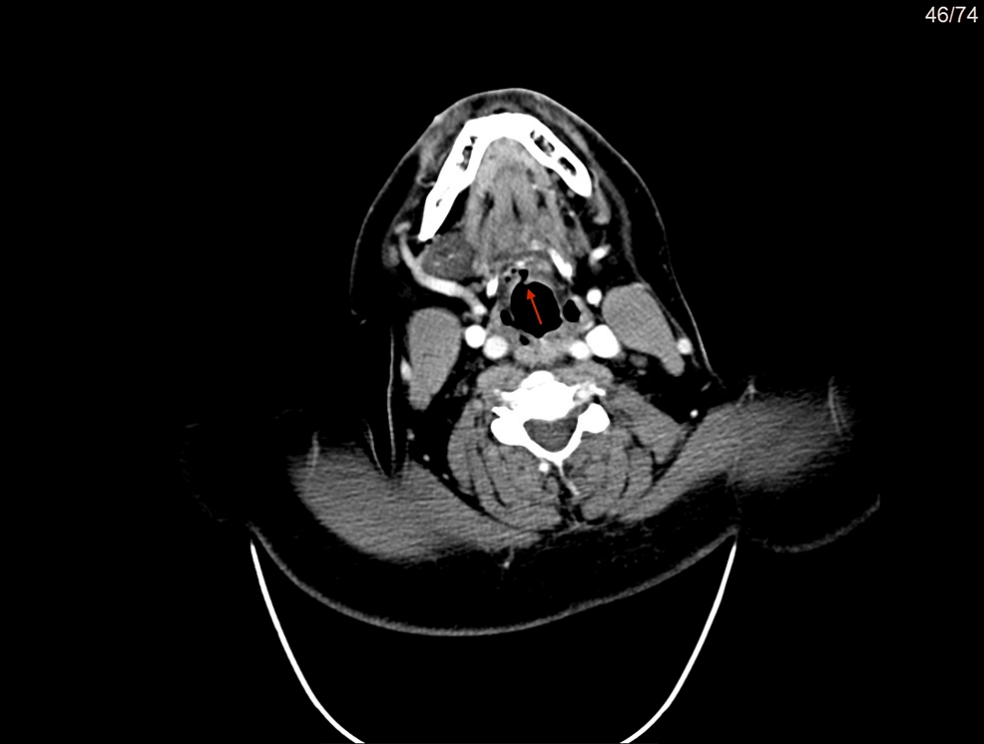
CT scan in axial view of the bifid epiglottis (red arrow)

**Figure 4:**
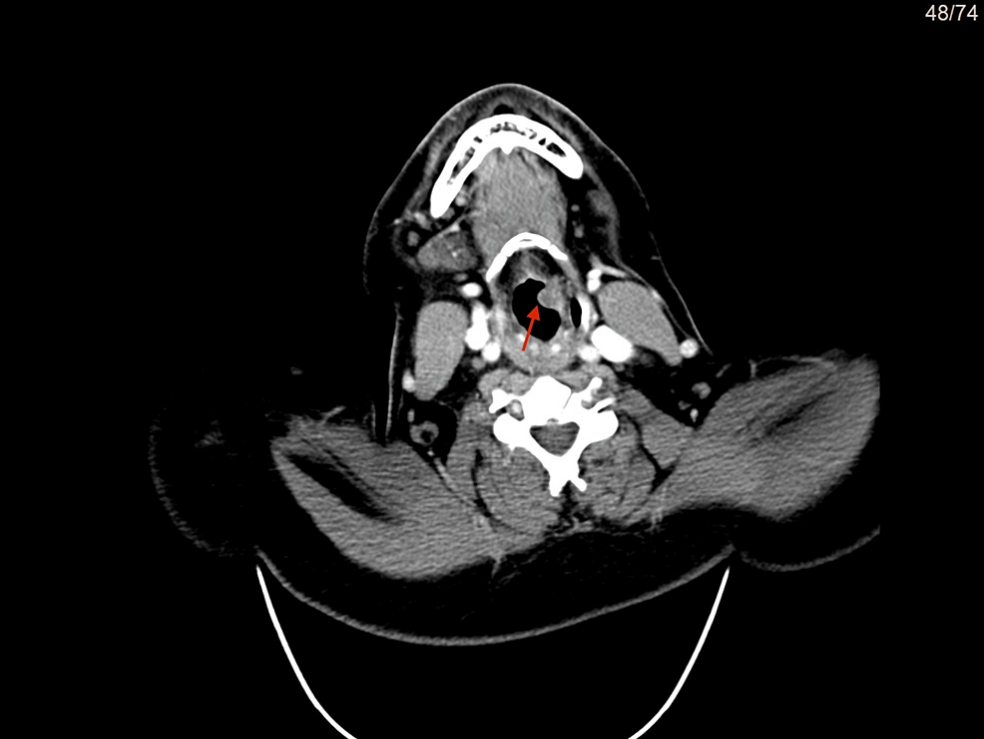
CT scan in axial view The red arrow shows thickening with a small polyp bulging at the left aryepiglottic fold with enlargement of the ipsilateral pyriform sinus.

**Figure 5:**
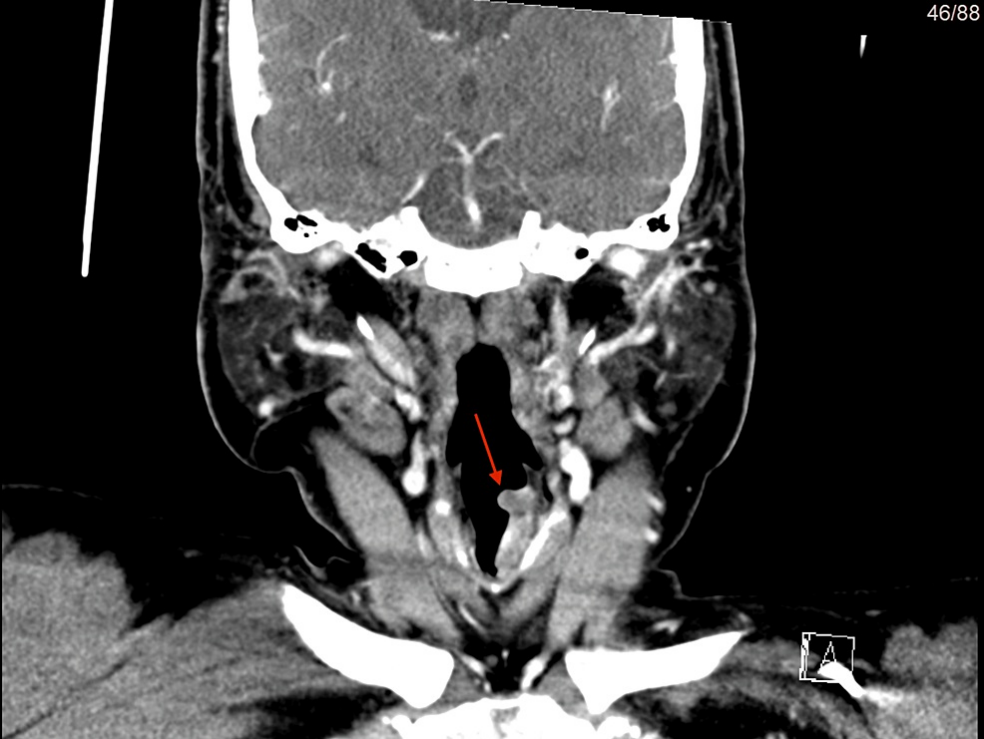
CT scan in coronal view of the mass shown by the red arrow.

A 25-year-old male known case of BBS was referred to the otolaryngology department for hoarseness of voice since birth; there was no change to his day-to-day life or change of diet. The patient did not complain of stridor dysphagia, dyspnea, gastroesophageal reflux, or other associated symptoms. Moreover, he reported experiencing asthma during childhood with multiple exacerbations due to recurrent upper respiratory tract infection, recurrent tonsilitis, loud snoring during sleep, and mouth breathing; for this, the patient underwent an adenotonsillectomy. On examination, he was noted to have craniofacial dysmorphism characterized by deep wide-set eyes, depressed nasal bridge and retrognathia, and postaxial polydactyly of the right hands and left foot. The family history showed that the parents were consanguineous (fourth-degree relatives), but they were phenotypically normal. The patient’s younger sister was also found to have BBS, which was confirmed by genetic testing. At initial ENT visits, fiberoptic nasopharyngolaryngoscopy (NPLS) revealed a laryngeal, pedunculated, bulging, rounded glottic mass and subglottic bulging with expiration and involuting with inspiration along with abnormal-looking epiglottis, having a separate cartilaginous framework with space in-between and bilateral mobile vocal cord. At the multidisciplinary team evaluation, the patient was found to be obese with a body mass index (BMI) of 44.5 (height, 157 cm; weight, 110kg), and also had an intellectual disability. The audiological exam was normal and no history of hyposmia or anosmia. An ophthalmologic exam revealed bilateral partial blindness due to retinitis pigmentosa associated with bilateral astigmatism. The cardiovascular, urological, and endocrine systems were all normal. Basic metabolic panel and complete blood count were within normal range; thyroxine (T4), thyroid-stimulating hormone (TSH), cortisol, follicle-stimulating hormone (FSH), and luteinizing hormone (LH) were normal. Computed tomography (CT) was done for this patient and showed the epiglottis which was bifid (Figures 1-3) and thickening with a small polyp bulging at the left aryepiglottic fold with enlargement of the ipsilateral pyriform sinus (Figures 4-5).

**Figure 6:**
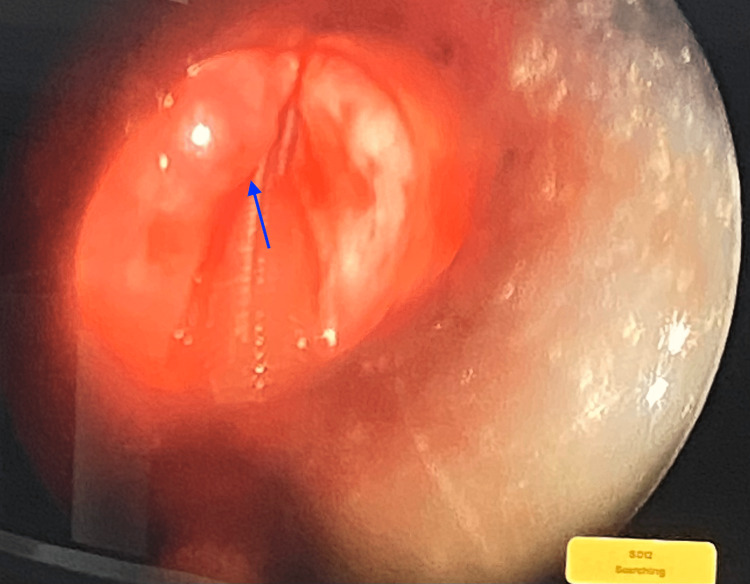
Micro-laryngoscopy view of the vocal cord mass that was excised endoscopically (blue arrow)

**Figure 7:**
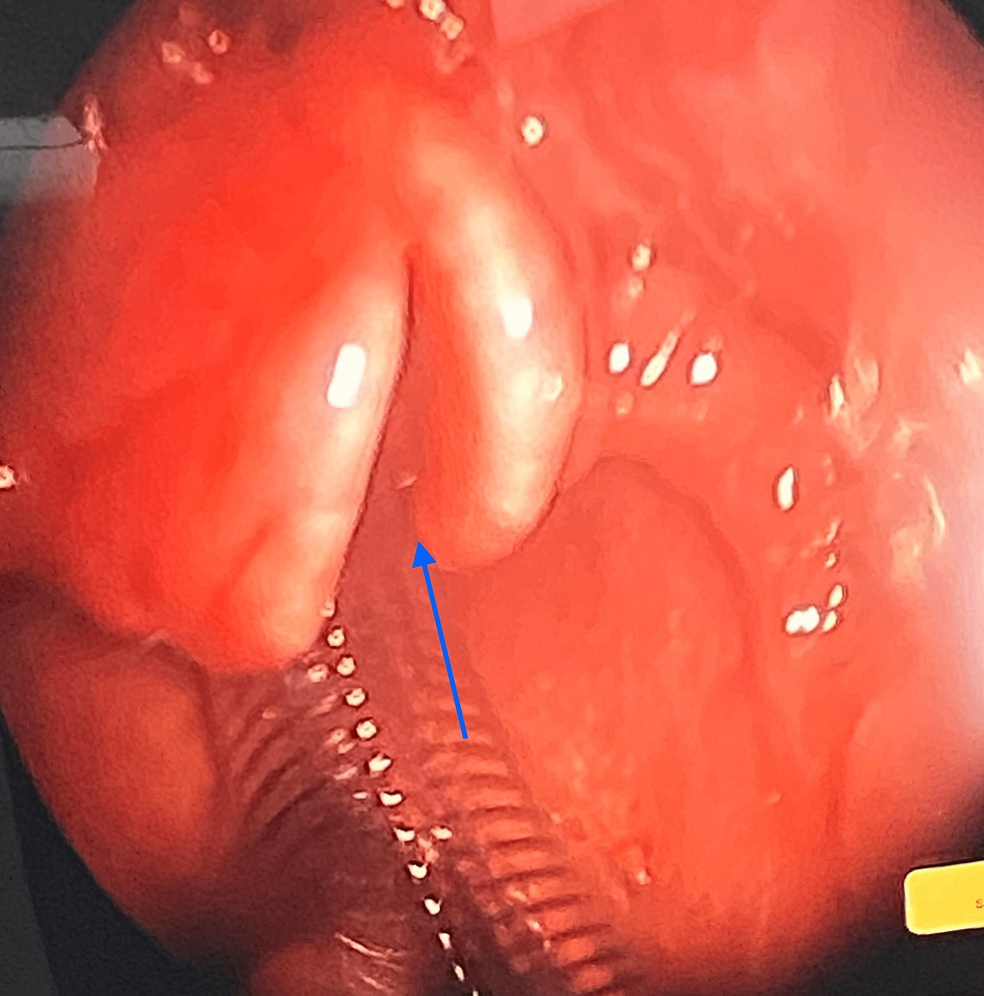
The microlaryngoscopy view shows bifid epiglottis, with the cleft reaching only the upper one-third, sparing the base (blue arrow).

The findings described could be related to left vocal cord paralysis, however, the possibility of underlying laryngeal tumor/chronic inflammatory process could not be totally excluded and required further evaluation by laryngoscopy. As a result, the decision was made to perform a micro-laryngoscopy and vocal cord mass excision under general anesthesia (Figure 6). Bifid epiglottis was observed as the cleft reaching the middle of the epiglottis did not reach the base (Figure 7). 

**Figure 8:**
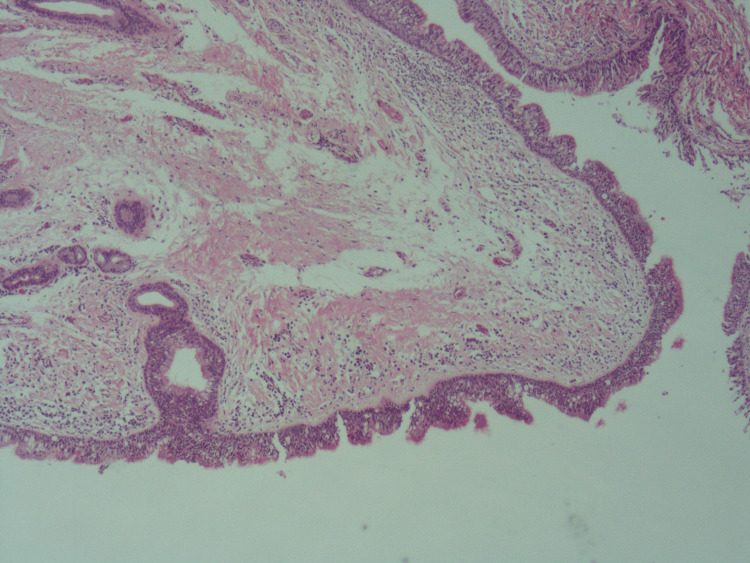
H&E stained slides at low power (4x) shows a polyp lined by unremarkable respiratory mucosa showing edematous stroma with telangiectatic blood vessels.

**Figure 9:**
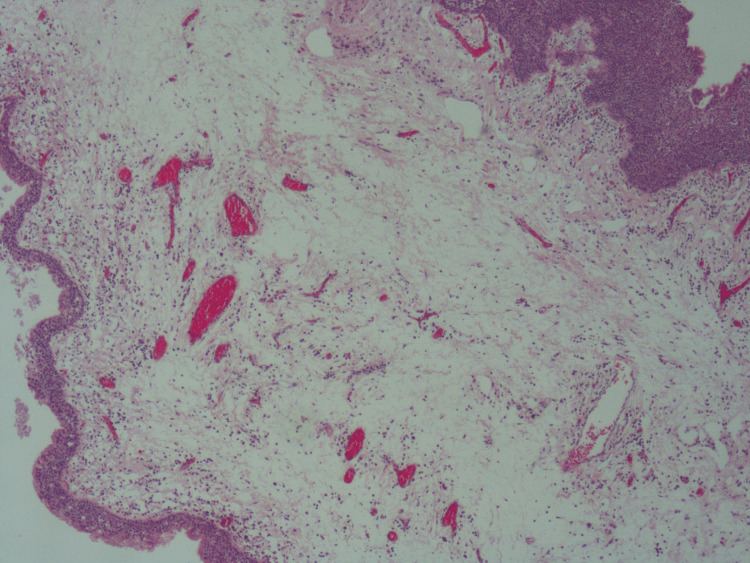
H&E stained slides at low power (4x) shows a polyp lined by unremarkable respiratory mucosa showing edematous stroma with telangiectatic blood vessels.

A vocal cord excisional biopsy was taken successfully, and patient recovery was uneventful. At the two-week follow-up, the patient showed improvement in the voice, and the biopsy results were unremarkable. The biopsy showed portions of respiratory mucosa with edematous stroma containing telangiectatic blood vessels and mid-lymphoplasmacytic inflammatory cell infiltrates and no dysplasia or malignancy (Figures 8-9).

## Discussion

The literature review is presented in Table 1.

**Table 1:** A literature review of all cases found from 1990-2022 of Bardet-Biedl syndrome associated with bifid epiglottis

Author (date)	Age/sex	Presentation	Scope findings	Management
Urben et al. (1999) [3]	10yr / M	Two episodes of respiratory distress due to croup required intubation, Frequent URTI	Bifid epiglottis, minimal suprahyoid development of the epiglottis, lateral pharyngoepiglottic folds were absent	Intubation due to acute respiratory distress (2 times)
Stevens et al. (2005) [5]	1yr / M	Chronic wheezing	Bifid epiglottis	Suturing of the epiglottis (another center) which was unsuccessful, gastrostomy tube placement and fundoplication resulted
Stevens et al. (2005) [5]	13½ yr / M	Loud snoring, sleep apnea	Bifid epiglottis	N/A
Chittoodan et al. (2010) [2]	8yr / F	Incidental finding of bifid epiglottis during intubation	Bifid epiglottis	N/A
Katsika et al. (2011 [6]	13yr / F	Incidental finding of bifid epiglottis during intubation for kidney transplant	Bifid epiglottis	N/A
Copennhaver et al. (2015) [7]	6yr / F	Recurrent pneumonia, wheezing	Bifid epiglottis and tracheobronchomalacia	Treated as a case of pneumonia and referred to ENT regarding bifid epiglottis
Cronjé et al. (2017) [8]	15 mo / M	Upper airway obstruction and stridor during anesthesia induction for club feet repair surgery	Bifid epiglottis with features of laryngomalacia, anterior laryngeal web, small right true vocal cord, shortened aryepiglottic fold, prolapsing right arytenoid cartilage	N/A
Poulin et al. (2019)[9]	9 mo / M	Dysphonia, recurrent respiratory infection, dysphagia, GERD	Bifid epiglottis, anterior laryngeal web	Conservative treatment for patient's laryngeal web
Kaur et al. (2021) [10]	11 mo / M	Noisy breathing and snoring, weak cry	Bifid epiglottis, anterior laryngeal web, membrane between vocal cords, small glottis chink	Laser excision of the glottic web was done under general anesthesia

BBS belongs to a group of ciliopathies and there is a clinical overlap between the different ciliopathies, which makes the diagnosis sometimes difficult. Usually, this syndrome is characterized by facial dysmorphism, post-axial polydactyly, retinal cone-rod dystrophy, obesity-related complications, short stature, cognitive impairment, renal anomalies, and genital anomalies. Bifid epiglottis is usually a syndromic constituent rather than an isolated anomaly [9].

The review of the literature from 1990 to 2022 reports 24 cases of bifid epiglottis, but only nine cases were associated with BBS [2-10] (Table 1), eight were not associated with a known syndrome but may manifest with different anomalies [4,11-16], one case was associated with Joubert’s syndrome [17], and six cases were associated with Pallister-Hall Syndrome [18-23]. We mainly focused on cases associated with BBS of which three cases were male and three were female. Unlike our case, most cases were in the pediatric age group ranging from nine months to 13 years. The most frequent presenting complaints of laryngeal abnormality in BBS included respiratory distress due to recurrent upper respiratory tract infection (URTI) and aspiration during feeding [3,7,9]. However, Stevens et al. [5] mentioned that it can present with snoring, obstructive sleep apnea, and wheezing, it can also present with dysphagia and gastric reflux symptoms [9]. Moreover, Chittoodan et al., Katsika et al., and Cronjé et al. [2,6,8] reported in their respective studies that bifid epiglottis could be completely asymptomatic. But in our case, the patient presented with hoarseness of voice. The onset of clinical manifestations is variable which makes the diagnosis difficult in some patients. Because of the multiple system involvement, a multidisciplinary approach is necessary with different medical specialties: pediatrics, neurology, dentistry, ophthalmology, endocrinology, ENT, genetics, cardiology, psychology, surgery, gastroenterology, and nephrology. Diagnosis is usually made with the help of endoscopy which could be flexible nasopharyngoscopy, direct laryngoscopy, or bronchoscopy in some cases [10,9]. In our case, initially, we performed flexible nasopharyngoscopy, then during the operation direct laryngoscopy was used. In terms of management, it depends mainly on the severity of presenting symptoms, conservative for asymptomatic patients as in most of the cases in our review, medical management for recurrent URTI with steroids, antibiotics, and bronchodilators [7], or surgical management by suturing the epiglottis [5], amputation, or in severe cases, tracheostomy. One case was associated with laryngeal web, so laser excision was performed [10]. In our patient, he was complaining of hoarseness due to vocal cord nodules after the excision of the nodules; in the follow-ups, the patient improved with no further intervention. 

## Conclusions

Although few reports focused on the presence of otolaryngologic abnormalities associated with LMBBS, to date, we found only nine cases that reported bifid epiglottis associated with BBS. Since the correlation between bifid epiglottis and BBS may be underestimated and rarely reported, further studies are required to confirm this correlation. Common presentations due to laryngeal abnormalities in patients with BBS include snoring, obstructive sleep apnea, wheezing, and dysphagia, as well as recurrent upper respiratory tract infections and aspiration during feeding. In the event of respiratory symptoms or swallowing abnormalities in a patient with BBS, an evaluation of the airway is indicated. When an otolaryngologist incidentally finds a bifid epiglottis, they should be aware that associated anomalies may exist in genetic syndromes, including LMBBS.
